# The New York Institute of Stomatology

**Published:** 1900-08

**Authors:** 

**Affiliations:** The New York Institute of Stomatology


### Reports of Society Meetings.

### THE NEW YORK INSTITUTE OF STOMATOLOGY.

A REGULAR meeting of the New York Institute of Stomatology was held Tuesday evening, March 6, 1900, at eight o'clock, at the office of Dr. William E. Hoag, 8 East Forty-third Street.

The minutes of the previous meeting were read and approved.

*The President.*—We have with us this evening, gentlemen, Dr. Learned, of Northampton, Mass., who will address the meeting upon "A New Method of inviting Sleep without Drugs."

(For Dr. Learned's paper, see page 462.)

*Dr. J. B. Learned.*—I cannot say too often that I hope by the end of this century we shall learn to sleep without drugs. Asking questions was to be a portion of this paper, and very likely the most important part of the exercise.

*The President.*—You will kindly accept Dr. Learned's invitation to take part in this exercise. It is doubtless a subject that interests us all.

*Dr. R. H. M. Dawbarn.*—Mr. President, I appreciate the courtesy of being asked to discuss the paper. It certainly brings to remembrance the importance of inviting sleep as much as possible.

We all know that when we are asleep the brain is more nearly devoid of blood than at any other time; and therefore anything that tends to draw blood from the brain to another part of the body is beneficial for this purpose. This is chiefly the explanation of the method we have just heard so lucidly explained. A plan that has stood me in good stead,—acting in a similar way,—and one that is much practised among our English friends, who as a nation are excellent sleepers, is the habit of having a late meal at, say, ten o'clock at night. It is so useful in this respect that in the course of nineteen years as a physician I have many hundreds of times advised it among my patients, and have never had occasion to regret it. Of course, I do not mean a hearty meal, but a cup of warm milk, bouillon, chicken or oyster broth, or anything of that simple sort, which will not require an effort for its digestion; and the results are most excellent. By this means the blood is drawn from the brain to the stomach, and accomplishes the same result as the physical gymnastics to which our attention has been called. Some take plain hot water, but there is not enough substance in that to effect the same purpose for a sufficient length of time, at least with most people. Those who have trouble in sleeping are often people who have from necessity had to do a good deal of night-work. If not from necessity, they have at least done some hard thinking shortly before retiring. Hence the brain-cells have been in a very active state of vibration, and with difficulty calmed down. Anything that tends to mental activity in the evening is a mistake for one who sleeps badly. Some people ought never to take a cup of coffee, even at breakfast; for the stimulus producing mental activity will be maintained even into the following night. This I have seen time and time again.

*Dr. Learned.*—Mr. President, I neglected to say that I sleep in the open air. In my own room, at the head of my bed, are two open windows, so that by stretching out either arm I can feel the moving air, for the windows are open three hundred and sixty-five nights in the year. In an adjoining room my son slept where the open air blew over him the whole night long, during all his early life. Accustomed to it, we ride in the open trolley with a brisk breeze at nine or ten o'clock in the evening in September as well as in the summer-time, and, well wrapped up, we all enjoy it. This is true in Northampton; I hope it is equally so in New York. I believe it is essential to have good air at night as well as in the daytime.

I was in the Tubercular Sanitarium at Rutland, Mass., recently, where the windows were kept open at night in this way. The patients were allowed to have them closed while they were preparing for bed, and when they were ready the windows were opened. Every bed was under an open window. There were two hundred tubercular patients. Open air, food, physical exercise, and sleep were the antitoxins there.

We have heard of the English mode of eating at night, and I wish we could practise it with as much impunity on this side, but the American people are a class of dyspeptics, and while I agree with you that a meal would draw the blood from the brain to the stomach, you will agree with me that large numbers of people would suffer from this treatment, already having eaten a surplus, and that a more valuable method would be to walk five or eight miles per day. All the physical powers are called into action by this means, and you are helping nature to perform better her part while the owner remains at his tasks in his normal and proper condition, so that, while I think it would be justifiable in some cases, it would not be in the majority.

*Dr. Dawbarn.*—Mr. President, I wish to say that I have recommended that method of promoting sleep any number of times, and have never known it to have bad results, but the very reverse of such. Of course, I do not mean a hearty meal, but a cup of warm milk or bouillon will not hurt one. I certainly wish to add that I do not think it at all new or theoretical, but a very old and very practical prescription. Here in New York it is standard advice in our profession.

*Dr. F. Milton Smith.*—Some time ago I heard a paper by Dr. Fillebrown, of Boston. He suggested at that time a remedy for sleeplessness, which was to confine the thought if possible, and if the thought could be confined one could go to sleep. He suggested that the word "sleep," repeated over and over, positively concentrating the mind on the one thought, would almost invariably bring relief. I have tried it for the past two years, almost invariably with success.

*The President.*—The next thing that should occupy us this evening is a paper by Dr. Allan, upon "Persistently Retarded Temporary Teeth."

(For Dr. Allan's paper, see page 509.)

The Secretary read the following letters:

### A SHORT SERIES ON DELAYED DENTITION.

### DISCUSSION OF DR. GEORGE S. ALLAN'S PAPER.

In compliance with Dr. Allan's request for information and data relative to practical cases of delayed permanent dentition and their treatment, I submit the following brief account of three cases, all of which differ widely in nature.

Case I., illustrated by lantern slide No. 1, is a case where the abnormally late development characterizes one tooth only, the others having erupted at about the normal time. It is the right superior cuspid of a boy aged seventeen. The deciduous cuspid is yet very firm, and the permanent is advancing on its dorsal aspect. There is apparently no good cause for the condition. The treatment is negative while waiting to see what nature will do in one year, when another radiograph will be taken.

Case II. represents a condition of delayed dentition of all the superior bicuspids, and cuspids and molars, illustrated by slide No. 2. The patient is a girl aged twelve, in good health. The incisors, superior and inferior, erupted at seven to eight years. She suffers a serious superior protrusion. A possible explanation for the delayed development is found in the history in that she was continuously ill from three and a half years of age to about seven. The ancestral history is wanting. The radiographs demonstrate the condition with great clearness. The treatment for the present will be to make radiographs frequently to ascertain the rapidity of development.

Case III. is very complicated. It is illustrated by Fig. 3. It represents two extremes of development side by side, besides a serious malposition long before eruption. The child, a girl, is eight and a half years of age, and suffers a serious superior retrusion, which obtains also in her father. Singularly the permanent cuspids of this side are almost completely erupted. The left side is perfectly normal. When she presented, about a year ago, she was shedding the first deciduous right superior molar by the natural eruption of what was supposed to be the first bicuspid, but which soon developed to be the cuspid. The deciduous cuspid anterior to it, which stood firmly, was immediately extracted. In the mean time the superior centrals and laterals were all protruded. The inferior arch developed so much faster than the superior that radiographs were taken to ascertain the cause, when it was found that on this right side only one bicuspid was developing, and it was seriously malposed, its long axis being about forty degrees out of its proper course, and it is rotated nearly half-way round. Its direction being inclined backward, it has advanced against the mesial surface of the first permanent molar, and is actually causing the absorption of the mesio-buccal root as seen by the radiographs. It is literally wedged between the cuspid and the first permanent molar, and if allowed to advance as best it could, its lingual-cusp would probably become quite firmly locked under the mesial marginal ridge of the molar.

This condition was discovered last November, and it was decided to see what nature could do to correct the condition, and accordingly three months' time, or nearly, was given between radiographs to determine this. On February 13 the radiograph seen to the left was taken under as nearly as possible the same conditions of tube, angles, and distance, all of which records are always accurately kept. As nearly as I can calculate, the bicuspid has advanced on the line of its long axis about eight-tenths millimetre, and has advanced to the surface but five-tenths millimetre. At this rate it would take probably four years for it to come to the surface. The extreme facial deformity caused by the superior retrusion requires prompt attention. Accordingly, the course being pursued is the extraction of the remaining deciduous molar and the adjustment of a very small jack between the first permanent molar and the cuspid, to force the latter forward to its proper position, and the anchoring of a very small device into the buccal cusp of the imprisoned bicuspid, and attached to the above jack, to extrude the locked bicuspid. This appliance is seen in position in Fig. 4.

Slide No. 5 represents a case of delayed bicuspids and cuspids at fourteen years. The permanent laterals have never formed.

Case I. (slide No. 1) is of a young lady seventeen years of age, with a serious depression of the superior arch and lip. The temporary cuspid is firmly in place, and has above it a deep fossa where the permanent cuspid would be expected to be. There is clinically every indication that the permanent cuspid has never formed. It lies palatally to the temporary cuspid, and its cusp is engaged against the lateral, which it has displaced somewhat. This condition has compelled the root to develop backward instead of the crown advancing.

Case VI. (slide No. 6) shows the contents of a tumor near the dome of the arch in the hard palate of a lady about twenty-five years of age. It had appeared only the last few years. The radiograph shows it to contain the missing permanent cuspid and second bicuspid. A series of several radiographs has shown these teeth to be located at about right angles to their proper position. As the arch is very much retracted, an effort will be made to put one or both of these teeth in their proper places in the arch.

In regard to the disturbing effects on his patient of the Röntgen rays, as very briefly reported to me by Dr. Allan, I must say that, while I know nothing of the condition of the tube or the length of exposure, I cannot believe they had anything to do with the symptoms reported. I have never seen or heard of any such effects. I would be glad to know more particulars.

Röntgen rays of weak penetration, such as would come from a low vacuum tube, would, if continued for, say, half an hour, or more, in close proximity to the body, possibly produce severe local disturbance in the form of a dermititis. Personally I have never seen a trace of general disturbance, and only a trace of local in just one case, as reported.

With kindest greetings to the members of The New York Institute of Stomatology,

Yours very fraternally,

WESTON A. PRICE.

CLEVELAND.

March 3, 1899.

DEAR DR. ALLAN:

I am very glad to send you a few negatives that I have made. Some are good, others indifferent.

Fig. 1 was taken to see if there was a permanent cuspid to come into the space where the temporary lateral had been lost a long time previous.

Fig. 2 was taken with a piece of bridge-work *in situ* to ascertain the strength of the root of the lateral, which is very slight compared with that of the cuspid.

Fig. 3. We wished to know if there was a permanent lateral. The negative shows that there is no lateral. The temporary cuspid and molars are in place, and the permanent cuspid and the two bicuspids can be clearly seen embedded in the jaw. The central is being thrown out of place somewhat, and it is caused by the permanent cuspid crowding against its root.

Fig. 4 was taken to find out the shape of the inferior wisdom-tooth, which is crowding against the second molar. The film was not put quite far enough back in the mouth to show the whole of it. The roots of the second bicuspid, first and second molars, with their canals and pulp-chambers, are beautifully distinct. I consider this a splendid negative.

I remain, very truly, yours,

Dwight M. Clapp.

From Dr. E. S. Talbot, Chicago:

### PERSISTENT TEMPORARY DEVELOPMENT.

BY EUGENE S. TALBOT, M.D., D.D.S., CHICAGO, ILL.

In dealing with the case of almost complete persistent temporary teeth at the age of fourteen, it must be remembered that there are several developmental factors to be considered. In the first place there is a tendency not marked but still observable for the periods of stress (which are indicated by dentitional phenomena) to appear later. In such cases the persistence of the temporary teeth would be an expression of advance. It has been observed that in such cases the temporary teeth remained nearly normal until removal was enforced by the appearance of the permanent teeth. In the case of a friend of mine every one of the temporary teeth remained until the thirteenth year, and would have remained longer had they not been removed. In other instances, molars have remained still later. This condition of belated periods of stress would be an expression of advance. It is possible, however, that the condition described may be an expression of the law of economy of growth, whereby an arrested development on one side resulted in an exaggerated development on the other. This condition may therefore be a reversion to the monophyodont (one set of teeth) of the lower vertebrata, whereby the dyphodont (two sets of teeth) is sacrificed to a more primitive type.

The monophydont condition implies an extra allowance of teeth, as Kollmann and Gegenbauer have shown. Besides the rudiments of the enamel organs for the milk teeth and permanent teeth, there are additional organs present in a very variable condition and number, nearer the external surface. They are, however, very generally present, and are exceedingly similar to the youngest stages of the normal enamel organs. Kollmann and Gegenbauer believe that they are abortive rudiments surviving from an ancestral condition in which teeth were more numerous. It is more probable that this may be the case, since there is evidence of hereditary defect. There is continually going on in the system a struggle for existence between different organs, and this struggle for existence, unless balanced by the nervous system, is apt to result in the gain of primitive structures at the expense of those later developed. These laws govern dental embryology as well as embryology in general. The question as to treatment would hence turn upon the fact whether this condition was an expression of advance like delayed periods of stress, or whether it was an expression of degeneracy under the law of economy of growth. In the latter case other stigmata of degeneracy would exist. It should be remembered also that proper development of the permanent teeth will turn upon, as Minot has pointed out, proper development of the dental shelf. This dental shelf is one of the latter development, so far as its relation for the provision for the permanent teeth is concerned. Interference from hereditary defect, or from causes operating during intra-uterine life, with the growth of the dental shelf would interfere with the growth of the permanent to the gain of the temporary teeth. The dental shelf (being of the transitory structure type), like all such, is more liable to atavistic tendencies than permanent structures.

Dear Doctor Allan,—The case of reported retarded eruption I spoke about was briefly as follows: Miss H., a child of twelve years, began to show signs of mental weakness. In one year she became idiotic, lost the use of words, could not care for herself in any way or manner, refused to wear clothing unless it was fastened so she could not remove it, lost all regard for her companions; and the family, after consultation, had concluded to send her to an institution. She would not eat, and she would try to get to her mother's breast to nurse. Her mother, with a mother's love and solicitude for the child, came and asked me to see her and give an opinion whether the trouble could come from the teeth, and, if so, could it be relieved; for the mother reasoned that the child could not eat or take food, or it would not want to go to her breast. With Dr. Blume I called and saw the child. No permanent teeth were in the mouth except the sixth-year molars, and they were all broken down. The gums were terribly inflamed, and bled at slightest pressure. Two of the temporary teeth only were missing. I advised the immediate extraction of the teeth, for I knew nature could not tolerate such a condition of congestion and inflammation as existed in this child's mouth without some reflex trouble. I was given *carte blanche,* and extracted *every* tooth, upper and lower. In three years the child was able to attend school, and to-day is in full possession of all her normal mental powers, and has permanent teeth in their position in the mouth.

LEVI L. HOWELL.

EAST HAMPTON, N. Y., June 11, 1899.

The case is a very interesting one, but I frankly own that I am unable to give any information that would serve to enlighten any one. The reason for the non-eruption of isolated teeth is quite clear, and the literature of dentistry is full of cases, but why an entire set should fail to follow the general law of development is not so clear to my mind. Dr. Guilford's case of the edentulous man is, possibly a near approach to this, and, possibly, could the X-rays have been used in the case, the germs of the permanent series would have been discovered.

I can no more explain this than I can explain why the whale should have a full series of mammalian teeth in fœtal life and balaen, or whalebone, subsequently, or why the so-called "bottle-nosed" whale should have a series of conical teeth in the front part of the jaw all completely hidden, besides rudimentary teeth.

We are yet in profound ignorance of the vital forces that control development, at least I am in the dark, and if Dr. Allan can divulge the reason in this particular case, I for one will be under lasting obligations to him.

Yours truly,

JAMES TRUMAN.

*Mr. E. W. Caldwell.*—The X-ray negatives which Dr. Allan has shown are made upon small pieces of sensitive film wrapped in such a way as to protect them from light and moisture and placed in the mouth of the subject, behind and as close as possible to the parts to be radiographed. The X-ray tube is placed from twelve to twenty inches from the patient's head and in such a position with reference to the film and the teeth to be examined that the shadow of the teeth on the film will suffer as little as possible from distortion. As

Dr. Allan has pointed out, it is not always easy to choose the best position of the tube for showing the parts correctly, and it is exceedingly difficult to make two pictures of the same part from exactly the same position. For this reason it is important to keep careful and accurate notes of the position of the tube with reference to the patient, so that if at a subsequent time another radiograph is needed, it can be made from the same position as the first one, and can therefore be safely compared with it.

I believe the best way of preparing the films for the mouth is to cover them with tissue-paper and place them between two layers of ordinary black dental rubber. The tissue-paper prevents the films from sticking to the rubber, and the black rubber, if carefully sealed around the edges, is sufficient to protect the films both from light and from the fluids of the mouth. In my first work of this kind (October, 1897) I used the ordinary cut films wrapped in black paper to exclude the light and sealed in envelopes of thin gutta-percha to protect it from the moisture of the mouth. The dental rubber makes a more flexible package than the gutta-percha and black paper, and it is much more convenient and easy to handle.

It is often desirable to expose more than one sensitive film at a time, and for this reason I prefer to use the thin "Kodak" films and place two or more in each envelope. The two or three films exposed at the same time may be developed separately, and by giving each one a different development it is often possible to bring out in each one some details that are lost in the others.

The X-ray tubes used for dental work should be of the highest possible efficiency. I prefer tubes of the large globular form, such as are made by the General Electric Company. The tubes I am using seem to give the best results when the vacuum is such that sparks will just pass between the leading-in wires when they are approached within about four inches of each other.

The duration of the exposure will depend upon the subject and the power of the tube. I have some good negatives with exposures of five seconds, but ordinarily I prefer to make an exposure of about half a minute with the tube fifteen to eighteen inches from the face.

With such exposures as this there is practically no danger of burning the patient, but when a great many radiographs are to be made within a few days it may be well to take the precaution to interpose between the tube and the patient a "grounded" metallic screen. This screen may be made of a piece of cardboard, about fifteen by twenty inches, covered with metal foil (gold-leaf or any similar material). The metallic surface of the screen should be grounded or connected to a gas- or water-pipe by means of a thin wire. It is not known just how effective this screen is in preventing burns, but it is safe and easy to use and probably affords some protection.

The X-ray tubes may be excited either by the discharge of an induction coil, a static machine, or a "high frequency" coil. An induction coil capable of delivering thick, "fat" sparks about ten inches long is probably the best and most reliable apparatus for the purpose. With an induction coil it is necessary to have a good interrupter. The ordinary vibrating interrupter usually supplied with an induction coil is not a very satisfactory machine, especially if the current is taken from electric lighting mains at about one hundred and fifteen volts. I prefer to use a rotary interrupter going about eighty breaks per second. I have used also electrolytic and liquid interrupters (New York *Electrical Review*, May 3, 10, 17, 1899) with very good results, but the rotary interrupter I am using is less noisy than the liquid interrupters, and is cleaner and more easily controlled. The Wehnett interrupter has the advantage of enabling an ordinary induction coil to be used for X-ray work with an alternating current supply.

In conclusion, I would suggest to those who contemplate fitting up an X-ray outfit to get the best apparatus to be had. At best it is laboratory apparatus, and the making of good radiographs requires the same patience and careful manipulation that are necessary in all difficult laboratory work.

*The President.*—Dr. Clapp, of Boston, will now exhibit some X-ray pictures of other cases. (Illustrations by means of screen pictures.)

*Dr. Dwight M. Clapp.*—Speaking of burns resulting from the X-ray, so far as I know the effect of burning will not be felt for about nine days after exposure. I have no means of knowing why. A gentleman exposed his little finger for about half an hour, at a distance of an inch from the tube, and he was most severely burned. It did not appear for about nine days, but it then began to show signs of trouble which was most persistent. It was a sore that did not yield to treatment. This was some three years ago, and his finger has not yet regained its natural skin and appearance. Burns seem to me to depend entirely upon the length of exposure and distance from the tube. I usually make three exposures of from one-quarter to one and one-quarter minutes. Good pictures can be obtained in about fifteen seconds. I have often made three exposures, and then in a few days made three more, and then again three more. I do not think it possible, at a distance of eight or ten inches, to burn with a fifteen-seconds exposure. It is necessary to be quite careful; although I have never had any trouble, others have. I have never seen any bad effects whatever. I should never hesitate to make the exposures.

*Dr. J. Morgan Howe.*—Before we adjourn I would like to move a vote of thanks to Dr. Learned for his interesting paper upon the subject of sleep, and to Dr. Clapp for his interesting and valuable exhibit and his explanation concerning radiographs.

Carried.

Adjourned.

Fred. L. Bogue, M.D., D.D.S.,
*Editor The New York Institute of Stomatology.*

